# Supplementary material for: The Small RNA Universe of Capitella teleta
Source: Front Mol Biosci. 2022 Feb 25;9:802814. doi: 10.3389/fmolb.2022.802814 (PMC8915122; doi:10.3389/fmolb.2022.802814)
Supplement: Supplementary file 1 [file DataSheet1.ZIP › Supplement/known/CAPTEscaffold_222_14880.pdf]

Provisional ID	: CAPTEscaffold_222_14880
Score total	: 32419.3
Score for star read(s)	: 3.9
Score for read counts	: 32408.7
Score for mfe	: 2.1
Score for randfold	: 1.6
Score for cons. seed	: 3
Total read count	: 63580
Mature read count	: 34997
Loop read count	: 0
Star read count	: 28583

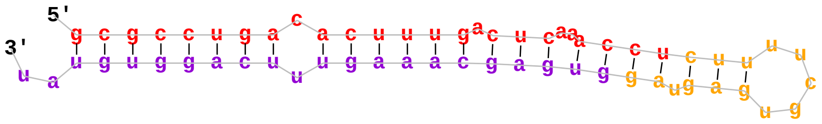

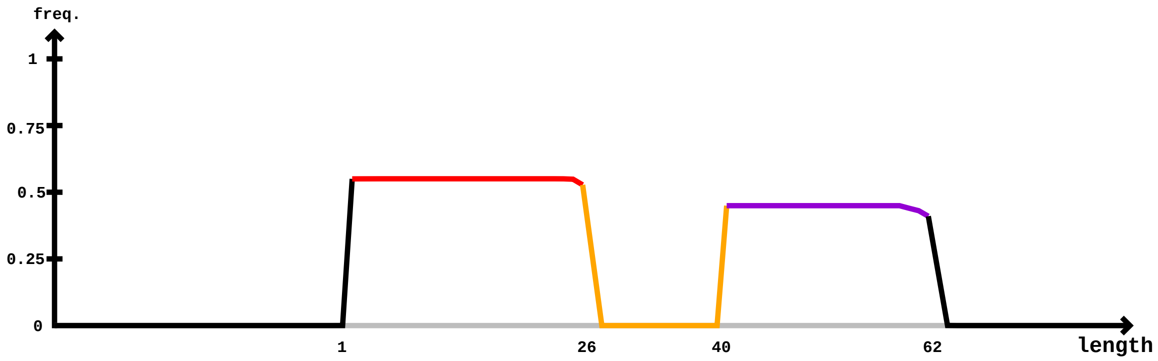

Mature Star

```
5'- uuaacgguggaguggaaugucgcgugguggcgccugacacuuugacucaaaccucuuuucgugaguaggugagcaaaguuucagguguaucugcgcgacugcauuaucaa  -3'   obs
    uuaacgguggaguggaaugucgcgugguggcgccugacacuuugacucaaaccucuuuucgugaguaggugagcaaaguuucagguguaucugcgcgacugcauuaucaa        exp
    uuaacgguggaguggaaugucgcgugguggcgccugacacuuugacucaaaccucuuuucgugaguaggugagcaaaguuucagguguaucugcgcgacugcauuaucaa        known
    .......(((((((....(((((((((..((((((((.((((((.(((..(((((((.....))).))))))))))))).))))))))..)))))))))..)))).))).     reads    mm    sample

    ...Gcgguggaguggaaugucgcguggug.................................................................................     1        1     seq
    ........................ugguggcgccugacacuuugacu...............................................................     1        0     seq
    ............................Cgcgccugacacuuugacucaaaccu........................................................     5        1     seq
    .............................gcgccugacacuuugacu...............................................................     1        0     seq
    .............................gcgccugacacuuugacucaa............................................................     3        0     seq
    .............................Ucgccugacacuuugacucaaa...........................................................     1        1     seq
    .............................gcgccugacacuuugacucaaa...........................................................     11       0     seq
    .............................gcgccugacacuuugacucaaac..........................................................     88       0     seq
    .............................gcgccuAacacuuugacucaaac..........................................................     1        1     seq
    .............................gcgccCgacacuuugacucaaacc.........................................................     1        1     seq
    .............................Ucgccugacacuuugacucaaacc.........................................................     3        1     seq
    .............................gcgccugacacuuugacucaaGcc.........................................................     1        1     seq
    .............................gcgccugacaAuuugacucaaacc.........................................................     1        1     seq
    .............................gcgccugacacuuugacucaaacU.........................................................     3        1     seq
    .............................gcgccAgacacuuugacucaaacc.........................................................     1        1     seq
    .............................gcgccugacacuuugacuAaaacc.........................................................     1        1     seq
    .............................gcgccugacacuuugacucGaacc.........................................................     1        1     seq
    .............................gcgccugacacuuugacucaaacc.........................................................     1328     0     seq
    .............................gcgccugCcacuuugacucaaacc.........................................................     1        1     seq
    .............................gcgccugacacuuAgacucaaacc.........................................................     1        1     seq
    .............................gcgccugaUacuuugacucaaacc.........................................................     1        1     seq
    .............................gcgccugacacuuugacucaaaUc.........................................................     2        1     seq
    .............................gcgccugacacuuugacucaGacc.........................................................     1        1     seq
    .............................gcgccugacacuuugacucaaaccu........................................................     32656    0     seq
    .............................gcgccuCacacuuugacucaaaccu........................................................     4        1     seq
    .............................gcUccugacacuuugacucaaaccu........................................................     6        1     seq
    .............................gcgccugaGacuuugacucaaaccu........................................................     4        1     seq
    .............................gcgccugacacuuugacucaaacUu........................................................     8        1     seq
    .............................gcgccugacacuuugacucaaaUcu........................................................     1        1     seq
    .............................gcgccugacacuuugacucaaaccG........................................................     118      1     seq
    .............................gcgccugacacuuugacucaGaccu........................................................     3        1     seq
```

Mature   Star

```
uuaacgguggaguggaaugucgcguggugcgccugacacuuugacucaaaccucuuuucgugaguaggugagcaaaguuucagguguaucugcgcgacugcauuaucaa
.............................gcgccugacacuuugacucaUaccu........................................................   3      1     seq
.............................gcgccugCcacuuugacucaaaccu........................................................   7      1     seq
.............................gcgccugacacuuugacuAaaaccu........................................................   2      1     seq
.............................gcgccugacacuuugacuGaaaccu........................................................   3      1     seq
.............................gcgccugacacuuugacCcaaaccu........................................................   1      1     seq
.............................gcgccugacacuuuAacucaaaccu........................................................   16     1     seq
.............................gcgUcugacacuuugacucaaaccu........................................................   2      1     seq
.............................gcgccugaUacuuugacucaaaccu........................................................   5      1     seq
.............................gcgcAugacacuuugacucaaaccu........................................................   5      1     seq
.............................gcgccugacacGuugacucaaaccu........................................................   1      1     seq
.............................Acgccugacacuuugacucaaaccu........................................................   5      1     seq
.............................gUgccugacacuuugacucaaaccu........................................................   3      1     seq
.............................gcgccugacacuuugacucaaCccu........................................................   5      1     seq
.............................gcgccugacacuuuCacucaaaccu........................................................   4      1     seq
.............................gcgccugacacuCugacucaaaccu........................................................   2      1     seq
.............................gcgccugacacuuAgacucaaaccu........................................................   10     1     seq
.............................gcgccugacacuuugacucaaGccu........................................................   4      1     seq
.............................Ncgccugacacuuugacucaaaccu........................................................   12     1     seq
.............................gcgccugacGcuuugacucaaaccu........................................................   5      1     seq
.............................gcgccugacacuuugacucCaaccu........................................................   1      1     seq
.............................gcgccugacacuuugaAucaaaccu........................................................   8      1     seq
.............................gcgccugacacuuugaUucaaaccu........................................................   9      1     seq
.............................gcgGcugacacuuugacucaaaccu........................................................   2      1     seq
.............................gcgccugacacCuugacucaaaccu........................................................   5      1     seq
.............................gcgccugacacuuugUcucaaaccu........................................................   7      1     seq
.............................gcgccugacacuuugacucaaaccA........................................................   145    1     seq
.............................gcgccugacacAuugacucaaaccu........................................................   9      1     seq
.............................gcgccugacacuuugacucaaacGu........................................................   1      1     seq
.............................gcgccugacaGuuugacucaaaccu........................................................   1      1     seq
.............................gcgccugUcacuuugacucaaaccu........................................................   5      1     seq
.............................gcgccuUacacuuugacucaaaccu........................................................   5      1     seq
.............................gcgcUugacacuuugacucaaaccu........................................................   7      1     seq
.............................gcgccugacacuuugacucaaacAu........................................................   12     1     seq
.............................gcgccugacacuuugacucaaaccC........................................................   5      1     seq
.............................gcgAcugacacuuugacucaaaccu........................................................   2      1     seq
.............................gcgcGugacacuuugacucaaaccu........................................................   1      1     seq
.............................gcAccugacacuuugacucaaaccu........................................................   6      1     seq
.............................gcgccugacacuuugaGucaaaccu........................................................   2      1     seq
.............................gcgccugacacuuugacuUaaaccu........................................................   16     1     seq
.............................gcgccugacaUuuugacucaaaccu........................................................   1      1     seq
.............................gcgccugGcacuuugacucaaaccu........................................................   6      1     seq
.............................gcgccugacaAuuugacucaaaccu........................................................   4      1     seq
.............................gcgccugacacuuCgacucaaaccu........................................................   7      1     seq
.............................gcgccugacacuuugacucaaaAcu........................................................   16     1     seq
.............................gcgccugacacuuugacGcaaaccu........................................................   1      1     seq
.............................gAgccugacacuuugacucaaaccu........................................................   12     1     seq
.............................Ucgccugacacuuugacucaaaccu........................................................   38     1     seq
.............................gcgccugacacuuugCcucaaaccu........................................................   6      1     seq
.............................gcgccugacacuuugacAcaaaccu........................................................   8      1     seq
.............................gcgccugacacuGugacucaaaccu........................................................   3      1     seq
.............................gNgccugacacuuugacucaaaccu........................................................   1      1     seq
.............................gcgccugacacuuugGcucaaaccu........................................................   7      1     seq
.............................gcgccugacacuAugacucaaaccu........................................................   8      1     seq
.............................gcgccugacacuuugacucGaaccu........................................................   16     1     seq
.............................gcgccuAacacuuugacucaaaccu........................................................   10     1     seq
.............................gcgccugacUcuuugacucaaaccu........................................................   5      1     seq
.............................gcgccGgacacuuugacucaaaccu........................................................   3      1     seq
.............................gcgccugacacuuugacucUaaccu........................................................   2      1     seq
.............................gGgccugacacuuugacucaaaccu........................................................   4      1     seq
.............................gcgccCgacacuuugacucaaaccu........................................................   11     1     seq
.............................gcgccugaAacuuugacucaaaccu........................................................   6      1     seq
.............................gcgccugacacuuugacucaaUccu........................................................   3      1     seq
.............................Ccgccugacacuuugacucaaaccu........................................................   13     1     seq
.............................gcgccAgacacuuugacucaaaccu........................................................   22     1     seq
.............................gcgccugacacuuugacucaaaccuA.......................................................   3      1     seq
.............................gcgccugacacuuugacucaaaccuc.......................................................   1      0     seq
.............................gcgccugacacuuugacucaaaccuU.......................................................   155    1     seq
..............................cgccugacacuuugacucaaacc.........................................................   1      0     seq
..............................cgccugacacuuugacucaaaccu........................................................   5      0     seq
..............................cgccugacacuuugacucaaaccuc.......................................................   5      0     seq
```

Mature Star

```
uuaacgguggaguggaaugucgcguggugcgccugacacuuugacucaaaccucuuuucgugaguaggugagcaaaguuucagguguaucugcgcgacugcauuaucaa
...............................gccugacacuuugacucaaaccu........................................................  8     0  seq
................................ccugacacuuugacucaaa...........................................................  1     0  seq
..................................ugacacuuugacucaaaccu........................................................  4     0  seq
....................................acacuuugacucaaaccu........................................................  1     0  seq
....................................................................gugagcaaaguuucaggu........................  4     0  seq
....................................................................gugagcaaaguuuUaggug.......................  1     1  seq
....................................................................gugagcaaaguuucaggGg.......................  2     1  seq
....................................................................gugagcaaaguuucaAgug.......................  1     1  seq
....................................................................gugaCcaaaguuucaggug.......................  1     1  seq
....................................................................gugagcaaaguuucagCug.......................  1     1  seq
....................................................................gugagcaaaguuucaggug.......................  598   0  seq
....................................................................guCagcaaaguuucaggug.......................  1     1  seq
....................................................................gAgagcaaaguuucaggug.......................  2     1  seq
....................................................................gugagcaaagAuucaggugu......................  1     1  seq
....................................................................gugagcaaaguuucaggugu......................  566   0  seq
....................................................................gugagcaaaguuucaggugG......................  14    1  seq
....................................................................gugagcaaaguuucaggugC......................  2     1  seq
....................................................................gGgagcaaaguuucaggugu......................  1     1  seq
....................................................................gugagcaaaguuuUaggugu......................  2     1  seq
....................................................................gAgagcaaaguuucaggugu......................  1     1  seq
....................................................................gNgagcaaaguuucaggugu......................  1     1  seq
....................................................................gugagcaaaguuucaAgugu......................  1     1  seq
....................................................................gugagcaaaguuucGggugua.....................  2     1  seq
....................................................................gugagcaaaguuucCggugua.....................  1     1  seq
....................................................................gugagcaaaguuucaggugua.....................  1293  0  seq
....................................................................gugagcaaaUuuucaggugua.....................  1     1  seq
....................................................................Uugagcaaaguuucaggugua.....................  4     1  seq
....................................................................gugagcaaaguuucagUugua.....................  1     1  seq
....................................................................guUagcaaaguuucaggugua.....................  1     1  seq
....................................................................gugagcaaaguuuUaggugua.....................  2     1  seq
....................................................................gugagcaaaguuucagguguC.....................  1     1  seq
....................................................................gugagcaaaguuucaggCgua.....................  1     1  seq
....................................................................gAgagcaaaguuucaggugua.....................  2     1  seq
....................................................................gugagcaaaguuucagguguU.....................  3     1  seq
....................................................................gugagcaaagCuucaggugua.....................  1     1  seq
....................................................................gugagcaaaguuucagguUua.....................  1     1  seq
....................................................................gugagcaaaguuCcaggugua.....................  1     1  seq
....................................................................gugagUaaaguuucagguguau....................  3     1  seq
....................................................................gugagcaaagAuucagguguau....................  5     1  seq
....................................................................gugCgcaaaguuucagguguau....................  1     1  seq
....................................................................gugaCcaaaguuucagguguau....................  2     1  seq
....................................................................guAagcaaaguuucagguguau....................  23    1  seq
....................................................................gugagcaaUguuucagguguau....................  1     1  seq
....................................................................gugagcaaaguuucaAguguau....................  15    1  seq
....................................................................gugagcaaagCuucagguguau....................  1     1  seq
....................................................................guCagcaaaguuucagguguau....................  4     1  seq
....................................................................gugagcaaaguuucagguguGu....................  436   1  seq
....................................................................gugagcaaaguuuUagguguau....................  57    1  seq
....................................................................Nugagcaaaguuucagguguau....................  7     1  seq
....................................................................gGgagcaaaguuucagguguau....................  6     1  seq
....................................................................gNgagcaaaguuucagguguau....................  1     1  seq
....................................................................gugagcaaaguuucCgguguau....................  1     1  seq
....................................................................guUagcaaaguuucagguguau....................  3     1  seq
....................................................................Cugagcaaaguuucagguguau....................  5     1  seq
....................................................................gugagcaaaguuucagguguaC....................  19    1  seq
....................................................................gugagcaaaguCucagguguau....................  3     1  seq
....................................................................gugagcaaaguuucagguAuau....................  2     1  seq
....................................................................gAgagcaaaguuucagguguau....................  17    1  seq
....................................................................gugaAcaaaguuucagguguau....................  2     1  seq
....................................................................gugagcaaaguuucagguguaA....................  25    1  seq
....................................................................gugagcaaaguuucaggugAau....................  3     1  seq
....................................................................gugagcaaaguuucagguguUu....................  6     1  seq
....................................................................gugagcaaaUuuucagguguau....................  2     1  seq
....................................................................gugagcaaaguuucaggGguau....................  3     1  seq
....................................................................gugaUcaaaguuucagguguau....................  2     1  seq
....................................................................gugagcaaaguuuAagguguau....................  4     1  seq
....................................................................gugUgcaaaguuucagguguau....................  2     1  seq
....................................................................gugagcUaaguuucagguguau....................  1     1  seq
....................................................................gugagcaaaguuucagguguaG....................  4     1  seq
....................................................................gugagcaaaguuucaUguguau....................  5     1  seq
```

```
                             Mature                                 Star
uuaacgguggaguggaaugucgcgugguggcgccugacacuuugacucaaaccucuuuucgugaguaggugagcaaaguuucagguguaucugcgcgacugcauuaucaa
....................................................................gugagcaaaguuucaggugCau....................	1	1	seq
....................................................................gugagcaUaguuucagguguau....................	1	1	seq
....................................................................gugagcaaagGuucagguguau....................	3	1	seq
....................................................................gugagcaaaguuucUgguguau....................	5	1	seq
....................................................................gugagcaGaguuucagguguau....................	1	1	seq
....................................................................gugagGaaaguuucagguguau....................	2	1	seq
....................................................................gugagcaaaguuucagUuguau....................	1	1	seq
....................................................................gugagcaaaguGucagguguau....................	3	1	seq
....................................................................gugagcaaaguuucagguUuau....................	4	1	seq
....................................................................gugagcaaaguuGcagguguau....................	2	1	seq
....................................................................Uugagcaaaguuucagguguau....................	32	1	seq
....................................................................gugagcaaGguuucagguguau....................	6	1	seq
....................................................................gugagcaaaguuucGgguguau....................	12	1	seq
....................................................................gugagcaaaguAucagguguau....................	10	1	seq
....................................................................gugagcaaaguuucagAuguau....................	3	1	seq
....................................................................gugagcGaaguuucagguguau....................	4	1	seq
....................................................................gugagAaaaguuucagguguau....................	8	1	seq
....................................................................Augagcaaaguuucagguguau....................	10	1	seq
....................................................................gugagcaaaguuuGagguguau....................	2	1	seq
....................................................................gugagcaaaguuAcagguguau....................	8	1	seq
....................................................................gCgagcaaaguuucagguguau....................	3	1	seq
....................................................................gugagcaaaguuucaggCguau....................	3	1	seq
....................................................................gugagcaaaguuucagguguau....................	23941	0	seq
....................................................................gugagcaaaguuucaCguguau....................	1	1	seq
....................................................................gugagcaaaguuucaggAguau....................	12	1	seq
....................................................................gugagcaaaguuucaggugGau....................	1	1	seq
....................................................................gugagNaaaguuucagguguau....................	1	1	seq
....................................................................gugGgcaaaguuucagguguau....................	1	1	seq
....................................................................gugagcaaaguuucagguguauU...................	1236	1	seq
....................................................................gugagcaaaguuucagguguauA...................	46	1	seq
....................................................................gugagcaaaguuucagguguauG...................	8	1	seq
....................................................................gugagcaaaguuucagguguauAu..................	2	1	seq
....................................................................gugagcaaaguuucagguguauUu..................	18	1	seq
.....................................................................ugagcaaaguuucagguguau....................	2	0	seq
.......................................................................agcaaaguuucagguguau....................	2	0	seq
........................................................................gcaaaguuucagguguau....................	2	0	seq
```
